# Using Social Media to Engage Justice-Involved Young Adults in Digital Health Interventions for Substance Use: Pilot Feasibility Survey Study

**DOI:** 10.2196/37609

**Published:** 2022-12-02

**Authors:** Anna Harrison, Johanna Folk, Christopher Rodriguez, Amanda Wallace, Marina Tolou-Shams

**Affiliations:** 1 Mental Health Service San Francisco Veterans Affairs Health Care System San Francisco, CA United States; 2 Department of Psychiatry and Behavioral Sciences Weill Institute for Neurosciences University of California San Francisco San Francisco, CA United States; 3 Department of Child and Adolescent Psychiatry Weill Cornell Medical College and Columbia University College of Physicians and Surgeons New York, NY United States

**Keywords:** substance use, young adult, social media, digital health technology, mobile phone

## Abstract

**Background:**

Young adults involved in the justice system have high rates of substance use disorders and low rates of treatment engagement. Most justice-involved young adults are supervised in the community—not incarcerated in jail or prison—where they have ongoing access to substances and experience significant barriers to care. When they do engage in treatment, they tend to have worse outcomes than justice-involved adolescents and older adults. Despite the need to develop targeted treatments, there are unique challenges in recruiting this population into clinical research. Digital health technology offers many novel avenues for recruiting justice-involved young adults into clinical research studies and disseminating substance use disorder treatments to justice-involved young adults. Because the vast majority of young adults regularly use one or more social media platforms, social media may offer a cost-effective and efficient way to achieve these goals.

**Objective:**

This study aimed to describe the process and feasibility of using social media platforms (Facebook and Reddit) to recruit justice-involved young adults into clinical research. Justice-involved young adults recruited from these platforms completed a survey assessing the acceptability of digital health interventions to address substance use in this population.

**Methods:**

Justice-involved young adults (aged 18-24 years) were recruited through paid advertisements placed on Facebook and Reddit. Participants responded to a web-based survey focused on their substance use, treatment use history, and acceptability of various digital health interventions focused on substance use.

**Results:**

A national sample of justice-involved young adults were successfully enrolled and completed the survey (N=131). Participants were racially diverse (8/131, 6.1% American Indian individuals; 27/131, 20.6% Asian individuals; 23/131, 17.6% Black individuals; 26/131, 19.8% Latinx individuals; 8/131, 6.1% Pacific Islander individuals; 49/131, 37.4% White individuals; and 2/131, 1.5% individuals who identified as “other” race and ethnicity). Advertisements were cost-effective (US $0.66 per click on Facebook and US $0.47 per click on Reddit). More than half (72/131, 54.9%) of the participants were on probation or parole in the past year and reported hazardous alcohol (54/131, 51.9%) or drug (66/131, 57.4%) use. Most of the participants (103/131, 78.6%) were not currently participating in substance use treatment. Nearly two-third (82/131, 62.6%) of the participants were willing to participate in one or more hypothetical digital health interventions.

**Conclusions:**

Social media is a feasible and cost-effective method for reaching justice-involved young adults to participate in substance use research trials. With limited budgets, researchers can reach a broad audience, many of whom could benefit from treatment but are not currently engaged in care. Proposed digital health interventions focusing on reducing substance use, such as private Facebook groups, SMS text message–based appointment reminders, and coaching, had high acceptability. Future work will build on these findings to develop substance use treatment interventions for this population.

## Introduction

### Background

Young adults exhibit high rates of substance use disorders (SUDs), yet few receive necessary SUD treatment. In the general population, 5.2 million (15%) young adults aged 18- 25 years reported SUD symptoms severe enough to warrant treatment [1]. Despite being more likely than older adults to meet the clinical criteria for a SUD, young adults are far less likely to receive treatment: only 331,000 (ie, 6.3% of those with an identified need) received specialty SUD services in the prior year [1]. Engaging young adults in SUD treatment can be challenging, as existing treatments often overlook the unique context and circumstances they face [2]. For example, young adults often initiate substance use at an earlier age and experience more psychiatric comorbidities than older adults [2-4]. They also face unique psychosocial barriers to treatment, such as unstable housing and finances [5]. Young adults also tend to have lower self-efficacy for abstinence and fewer coping skills, both of which are key to SUD treatment success [6]. As many young adults have peers who are also using substances [1], their social context tends to be less conducive to reducing use or abstaining. Moreover, popular mutual help organizations, such as Alcoholics Anonymous, may be less appealing to young adults: only 13% of Alcoholics Anonymous members are aged ≤30 years [7].

Substance use problems are particularly common among young adults involved with the justice system [8], with recent national estimates showing that 39% of young adults (aged 18-25 years) on probation and 41% on parole or supervised release meet the clinical criteria for a SUD [9]. Justice-involved young adults have greater difficulty accessing health care than their older counterparts (eg, because they are more likely to remain uninsured [10]). Involvement in the justice system may exacerbate challenges young adults already face with treatment access and engagement. Of the approximately 6 million adults involved with the US adult correctional systems on any given day, approximately 4.5 million live in the community either on probation or parole [11] with ongoing access to substances. Justice-involved young adults are also more likely to live in urban neighborhoods with a disproportionately high density of outlets selling substances (eg, liquor stores and marijuana dispensaries), which may trigger cravings or lead to increased substance use and relapse [12,13]. In response, community supervision agencies across the country have increasingly incorporated SUD treatment into community corrections [14]. Diversion programs offering alternatives to incarceration for those charged with drug offenses are becoming more common, and evidence suggests they are effective at reducing both substance use and recidivism [15]. Specialty drug courts, a subset of collaborative courts that typically mandate participation in SUD treatment, have also proliferated in recent years [16], thus increasing access to SUD treatment.

Despite the criminal justice system’s embrace of these practices, uptake of SUD treatment for the broader community-supervised population has been slow [17]. Only approximately one-third of justice-involved adults with SUD receive treatment in any given year [10]. Significant barriers to treatment engagement may include stigma as well as a failure to identify substance use as a problem behavior [10]. When referred to SUD treatment by probation, young adults are less likely to identify their substance use as a problem behavior that requires change, despite experiencing associated legal consequences, compared with older adults [4]. Furthermore, justice-involved young adults tend to have worse treatment outcomes than both adolescents and older adults [2].

New strategies are needed to reach justice-involved young adults and improve their access to SUD treatment tailored to their unique circumstances. Digital health technology offers numerous ways to adapt SUD treatment to the specific needs of justice-involved young adults. Ranging from web-based to SMS text messaging to smartphone app, digital health technology can facilitate rapid delivery of evidence-based interventions [18]. For example, SMS text messaging interventions have led to tobacco use reductions in young adults [19]. A recent meta-analysis revealed that in the primary care setting, SMS text messaging interventions improve retention in treatment and medication adherence and promote reductions in alcohol, methamphetamine, and opioid use. Web-based interventions are comparable with in-person interventions in terms of their success in improving treatment engagement and increasing abstinence [20].

Despite its promise, to our knowledge, no studies have examined digital health interventions developed specifically for justice-involved young adults. Although digital health technology has shown promise among the adult probation population [21,22], to our knowledge, the unique needs of young adults have not been specifically explored or addressed. More research is needed about how to test and scale substance use interventions using technology with justice-involved young adults. Little is known about their preferences for the format of digital health interventions to address substance use. The first step is to see if and how to recruit justice-involved young adults into such research.

In total, 84% of all young adults (aged 18-29) in the United States use one or more social media platforms [23]. Even populations of young adults without reliable internet access, such as those experiencing homelessness, report using social media regularly [24]. Social media, particularly Facebook, has been effective for recruiting other subpopulations of young adults (such as smokers and military veterans) into digital health interventions [25-27]. Social media has also been shown to be an effective platform to recruit hard-to-reach populations and traditionally marginalized populations, such as HIV-positive men who have sex with men, Spanish-speaking Latino gay couples, and others [28-32]. Facebook is also an effective medium to deliver interventions [33]. Young adults who use Facebook are diverse in regard to racial and ethnic backgrounds, socioeconomic statuses, and geographic locations. Thus, Facebook may be one way to both recruit justice-involved young adults and deliver digital health SUD interventions specific to substance use. Other social media platforms have similar user bases and promise for recruitment.

### Objectives

This pilot feasibility study seeks to understand (1) if and how social media might be used to effectively reach justice-involved young adults for digital health substance use intervention trials and (2) what types of digital health interventions targeting substance use would justice-involved young adults be willing to participate in when such interventions are eventually brought to scale. This study lays the groundwork for future research adapting digital health interventions for this underserved population, thus expanding access and engagement in SUD treatment.

## Methods

### Participants

Participants were 131 young adults from across the United States. To be eligible, participants needed to (1) be aged between 18 and 24 years, (2) have been arrested within the past year, (3) have access to a computer or mobile device with the internet, and (4) be proficient in English. Respondents were not eligible if they were currently incarcerated.

### Procedures

**Figure 1:**
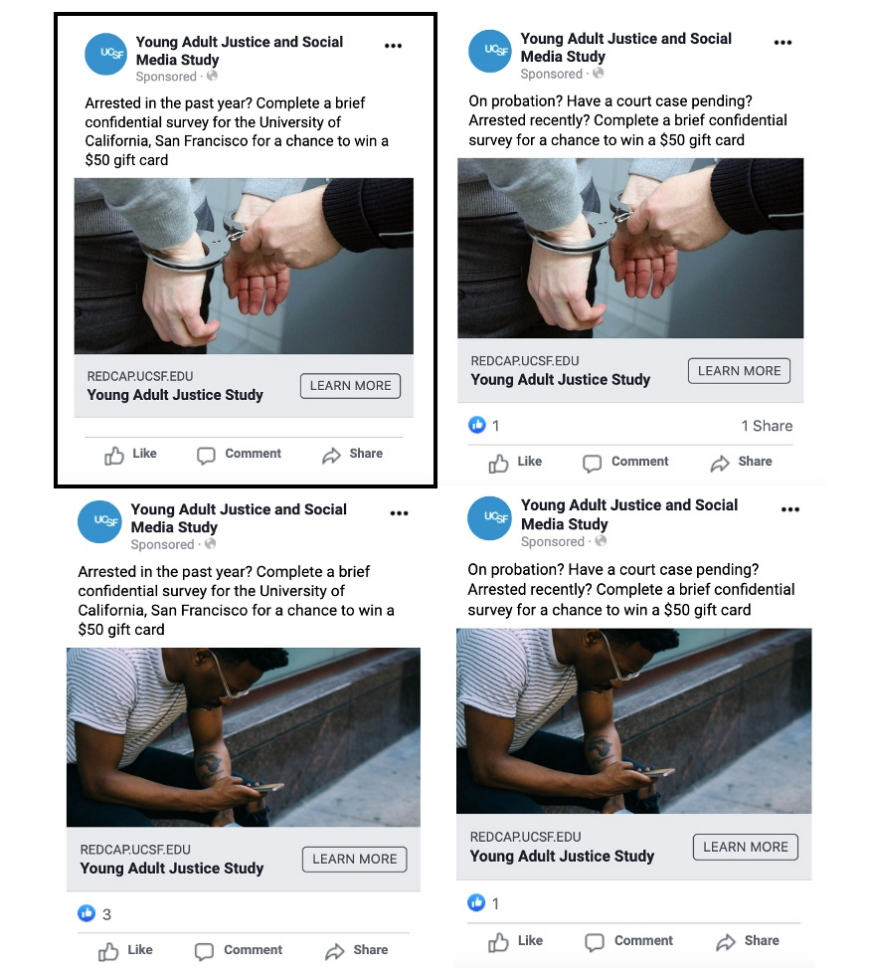
Facebook advertisements used for pilot testing. Black outline signifies the most cost-effective advertisement.

Participants were recruited through Facebook and Reddit paid advertisement campaigns (Figure 1). These platforms were selected because of their popularity among young adults: according to Pew Research Center, 70% of adults aged 18-29 years use Facebook and 36% use Reddit [23]. We included both these platforms to minimize sampling bias, as their intended uses and user bases tend to be somewhat different. Moreover, Reddit has specific groups (“subreddits”) for users involved in the justice system, increasing the advertisement’s visibility to the study’s target audience. All study advertisements were direct promotions for the survey website, such that individuals who clicked on the advertisement would be immediately directed to an external website (REDCap [Research Electronic Data Capture; Vanderbilt University]) containing a screening questionnaire for the web-based survey. Eligible participants were then directed to a study information page outlining the purpose of the study, contact information for the principal investigator, and the consent form. We also maintained a Facebook page where participants could review this material, which was reachable from the advertisements themselves. After providing informed consent, respondents were directed to the web-based survey. Participants who submitted their survey and who met eligibility criteria received the incentive. A total of 145 respondents were deemed eligible and completed the consent document. Of the 145 participants, 8 (5.5%) subsequently indicated they had no criminal justice contact in the past year (despite responding “yes” on the screening questionnaire) and 6 (4.1%) did not respond to any questions on the survey. These participants were removed from the final analytic sample. In 2 cases, participants entered the same email address for reimbursement; in these cases, the second response was removed.

### Advertising Pilot-Testing

#### Overview

Study advertising was conducted in an iterative manner. We used the Facebook A/B split testing feature in the advertising manager to compare 2 different images paired with 2 different text options. The A/B split testing feature shows different advertisements to similar types of Facebook users to understand which advertisements garner the most attention. We purposefully chose 2 images with very different stimulus values and paired each of the images with 2 different text options with the aim to better understand the unique effects of both the image and text. These 4 unique advertisements were initially deployed in the advertisement manager to understand which combination of image and text generated the most engagement with our target population (Figure 1).

The 4 different test advertisements ran for 3 days, from August 30, 2018, to September 2, 2018. At the end of the 3-day trial period, 1 advertisement was determined to be the most “successful” (ie, received the most unique link clicks at the lowest cost per click). After the initial A/B split testing was complete, advertisement campaigns using the most successful advertisement were launched through both the Facebook and Reddit paid advertising platforms.

In addition to the paid advertising campaigns, we posted the images and text in relevant Facebook groups (eg, Probation Researcher Network, SF Adult Probation, and Surviving Probation) and on subreddit threads (eg, Probation, Criminal Justice, Jail, Prison, and Paid Studies) at no cost. These appeared as regular posts and were not displayed as advertisements. As the autofiltering function in Reddit flagged our posts in subreddits as spam, the text of these advertisements was posted with no accompanying image.

During this initial advertisement launch, the incentive was an entry into a raffle for a US $50 gift card. Although these advertisements had satisfactory engagement on social media (on Facebook, the initial launch garnered 56,441 impressions and 244 clicks over the course of 25 days, which came to US $0.67 per click; on Reddit, there were 165,988 impressions with 733 clicks over the same period for US $0.42 per click), they yielded few participants. For example, the original Facebook advertisements yielded 24 people who completed the screener, with 11 eligible, 4 consenting, but only 3 completing the survey. From the original Reddit advertisements, 50 people completed the screener, with 10 eligible, 7 consenting, but only 6 completing the survey. Given these low enrollment numbers, we streamlined our study information sheet and increased our incentive to be a guaranteed US $10 gift card. Using the same winning advertisement from the original A/B split test, with the text updated to reflect the new incentive, advertisements were relaunched and posted in the same relevant Facebook groups. The sample for this study included only participants from the final advertisement strategy approach (ie, updated study information sheet and increased incentive).

#### Facebook Advertising Strategy

Paid advertisements were optimized for link clicks and presented to adults aged 18-24 years who used the platforms in English in the 10 most populous metropolitan areas in the United States (New York, Newark, or Jersey City; Los Angeles, Long Beach, or Anaheim; Chicago, Naperville, or Elgin; Dallas, Fort Worth, or Arlington; Washington, Arlington, or Alexandria; Houston, The Woodlands, or Sugar Land; San Francisco, Oakland, or Hayward; Philadelphia, Camden, or Wilmington; Boston, Cambridge, or Newton; and Atlanta, Sandy Springs, or Roswell). As our advertising budget was limited, we chose to limit advertisements to densely populated urban areas where community-supervised young adults are the most likely to reside. Advertisements ran over 12 days (November 11, 2018, to November 23, 2018) with a budget of approximately US $55 per day for a total of US $587.74.

#### Reddit Advertising Strategy

The advertising strategy for Reddit was as similar as possible to the strategy deployed for Facebook; however, there were some alterations because of inherent differences in the advertisement managers. Advertisements were optimized for link clicks and presented to users in the following 11 metropolitan areas, which corresponded as closely as possible to options in the Facebook advertisement manager (noted earlier): New York, Los Angeles, Chicago, Dallas, Washington, District of Columbia, Houston, San Francisco/Oakland/San Jose, Philadelphia/Wilmington, Boston/Manchester, and Atlanta. The advertisement manager did not allow for targeting of specific age demographics. Although it does allow for targeting of specific groups, or subreddits, the subreddits of potential interest (eg, criminal justice) to our population were not able to be targeted because of their small sizes. Advertisements ran over 14 days (November 11, 2018, to November 26, 2018) with a budget of approximately US $55 per day and a bid cap per 1000 impressions of US $1.00 for a total of US $786.26.

### Measures

#### Demographics and Criminal Justice Characteristics

Participants completed a brief demographics questionnaire to identify their gender, race, ethnicity, marital status, and current criminal justice involvement (eg, currently on probation or parole)

#### Substance Use

The *Alcohol Use Disorders Identification Test* (AUDIT [34]), a 10-item self-report screening tool, was used to assess past-year alcohol consumption, drinking behaviors, and alcohol-related problems. Responses are rated on a 0-4 scale (anchors differ for each question), with a maximum score of 40 and higher values representing more problematic alcohol use. Individuals who score ≥8 are considered at risk for or experiencing alcohol problems [35]. There is considerable evidence for the AUDIT’s internal consistency [36,37] and validity [34,38,39]. In this study, internal consistency was 0.89.

The *Drug Use Disorders Identification Test* (DUDIT [40]), an 11-item self-report assessment designed to parallel the AUDIT, was used to assess drug-related problems. Responses are rated on a 0-4 scale (anchors differ for each question), with a maximum score of 44; males with scores of ≥6 and females with scores of ≥2 are considered to probably have drug-related problems [40]. The DUDIT is psychometrically sound, with high internal, convergent, and discriminant validity [41]. In this study, internal consistency was 0.95.

#### Substance Use Treatment

Participants self-reported whether they were currently participating in substance use treatment and if so, what type of treatment (eg, residential treatment, outpatient treatment, or peer support groups), whether it was court mandated, and the proportion of scheduled sessions they typically attend. Participants also rated their level of motivation to participate in treatment (1=*not motivated at all* to 5=*very motivated*).

#### Acceptability of Digital Health Interventions

Participants reported their access to electronic devices (eg, smartphone and computer) and their willingness to participate in digital health interventions focused on substance use (1=*I would definitely participate* to 5=*I would definitely not participate*). Participants were asked to identify whether they would participate in any of the following programs (yes or no) and to rate which top 3 sound most interesting to them: (1) receiving appointment reminders via SMS text messages, (2) web-based peer support community, (3) private groups on Facebook focusing on reducing substance use where you can connect with peers, (4) private groups on Facebook focusing on reducing substance use where you can connect with peers and chat or contact a provider, (5) motivational posts on social media (eg, Instagram or Snapchat), (6) sessions with providers through video chat (eg, Facetime or Skype), (7) coaching by providers through SMS text messaging or secure messaging, and (8) others (please specify).

### Informed Consent and Participation Incentive

The consent form outlined the risks and benefits of study participation and confidentiality procedures. Participants were informed that the information would not be provided to court staff, police, or other justice system staff and that participation did not have any bearing on court-, probation-, or parole-related program requirements. As an incentive for participation, survey respondents were offered a US $10 Amazon gift card delivered via email. Participants were also provided with contact information of the principal investigator. After completing the survey, participants entered an email address, which was disconnected from survey responses. All study data used in analyses were anonymous.

### Ethics Approval

All study procedures were approved by the University of California, San Francisco, Institutional Review Board (IRB approval number: 18-24899).

### Plan of Analysis

We first describe findings from pilot advertisement and discuss how these data informed final advertisements to recruit the analysis sample. We then present demographic characteristics, criminal justice involvement, substance use, and treatment characteristics for participants from each recruitment platform. Participants who did not respond to all questions on the AUDIT (n=27) or the DUDIT (n=16) were excluded from those analyses. There were 6 participants who did not respond to items about current treatment and 9 who did not respond to items concerning the acceptability of digital health interventions. As this was a pilot study focused on the feasibility of recruiting a sample using social media, we did not conduct hypothesis testing to examine demographic or other differences for each platform or use advanced statistical methods to impute missing data. Differences in treatment preferences for those with and without hazardous substance use were assessed using independent sample *t* tests (2-tailed).

## Results

### Cost-effectiveness of Facebook and Reddit Advertisements

During the initial A/B split testing phase, the 4 “test” advertisements shown in Figure 1 were shown to a total of 44,168 people, resulting in 246 unique link clicks and costing US $164.96. Cost per click varied by advertisement. The most successful advertisement was an image of a White person’s hands being handcuffed with accompanying text, “Arrested in the past year? Complete a brief confidential survey for the University of California, San Francisco for a chance to win a $50 gift card.” This advertisement cost US $0.57 per click. The least successful advertisement, an image of a young Black man using a cellphone in an urban environment with the same text, cost US $0.76 per click.

Facebook advertisements were shown to a total of 90,431 Facebook users, with a total number of 818 unique link clicks. The cost per click was US $0.66. In total, we recruited 37 young adults through paid Facebook advertisements, which translated to an advertising cost of US $15.88 per participant. No participants were successfully enrolled from Facebook group posts (which were posted at no cost).

Reddit advertisements were shown to a total of 401,895 Reddit users, with a total number of 1618 advertisement clicks. The cost per click was US $0.47. In total, we recruited 94 young adults and spent a total of US $768.26 on Reddit advertisements, which translated to an advertising cost of US $8.17 per participant. A total of 3 participants were successfully enrolled from subreddit posts (which were posted at no cost).

### Sample Characteristics

In total, 37.4% (49/131) of the sample identified as White, and the majority of the sample (72/131, 59%) identified as cisgender men (Table 1). The median (IQR) age was 21 (20-23) years. Participants were largely single (94/131, 77.7%) and reported a wide range of experiences with the criminal justice system. In the past year, most (84/131, 64.1%) participants spent one or more days in jail, and 20% (26/131) of the participants spent one or more days in prison (Table 2). Approximately 40.5% (53/131) of the participants had been on probation in the past year, and 16.9% (22/131) of the participants had been on parole. Moreover, 12% (15/131) of the sample reported involvement with collaborative courts in the past year.

Both alcohol and drug use were common. The mean AUDIT score was in the “hazardous” range at 10.8 (SD 10.1), and just more than half (54/104, 51.9%) of the participants scored in the “hazardous” range. Similarly, the mean DUDIT score was 12.3 (SD 12.5), and more than half (66/115, 57.4%) of the participants scored in the hazardous range. Nearly one-fourth (28/125, 22.4%) of participants reported current participation in substance use treatment, and the vast majority (26/28, 96%) of the participants reported treatment that was mandated by the court. The most common treatment modalities were peer support groups (such as Alcoholics Anonymous, Narcotics Anonymous, and Life Ring; n=16); individual counseling (n=14); and group counseling (n=12). Of those who were engaged in formal individual or group therapy, most (22/28, 79%) reported attending regularly (more than 75% of the sessions). Those who reported alcohol or drug use in the hazardous range (n=85) placed greater importance on reducing substance use compared with those without hazardous substance use (n=18; t_101_=−7.31; *P*<.001). There were no significant differences between groups in participants’ confidence in their ability to reduce substance use.

We did not conduct hypothesis testing to detect differences in participant characteristics between recruiting platforms. It is notable, however, that Facebook appeared to be somewhat more efficient in recruiting Hispanic or Latinx participants: approximately 30% (11/37) of the participants recruited through Facebook identified as Hispanic or Latinx, whereas only 16% (15/94) of the participants recruited through Reddit identified as such. Regarding recent incarceration history, approximately 90% (33/37) of the participants recruited via Facebook had been incarcerated in jail in the past year, whereas only a little more than half (51/94, 54%) of the Reddit users reported jail time in the past year. Electronic monitoring appeared to be more common among Reddit users (32/94, 34%) than Facebook users (5/37, 14%).

**Table 1:** Study sample demographics.

		Total sample	Facebook	Reddit
**Gender (total sample: n=122; Facebook: n=34; Reddit: n=88), n (%)**				
	Cisgender woman	36 (29.5)	8 (23.5)	28 (31.8)
	Cisgender man	72 (59)	22 (64.7)	50 (56.8)
	Transgender woman	5 (4.1)	0 (0)	5 (5.7)
	Transgender man	3 (2.5)	2 (5.9)	1 (1.1)
	Nonbinary	5 (4.1)	2 (5.9)	3 (3.4)
	Other	1 (0.8)	0 (0)	1 (1.1)
Age (years), median (IQR)		21 (20-23)	21 (20-23)	22 (21-23)
**Age (years; total sample: N=131; Facebook: n=37; Reddit: n=94), n (%)**				
	18-19	23 (17.6)	12 (32.4)	11 (11.7)
	20-21	44 (33.6)	10 (27)	34 (36.2)
	22-24	64 (48.9)	15 (40.5)	49 (52.1)
**Race and ethnicity^a^ (total sample: N=131; Facebook: n=37; Reddit: n=94), n (%)**				
	Latinx or Hispanic	26 (19.8)	11 (29.7)	15 (16)
	American Indian	8 (6.1)	0 (0)	8 (8.5)
	Asian	27 (20.6)	6 (16.2)	21 (22.3)
	Black or African American	23 (17.6)	6 (16.2)	17 (18.1)
	Pacific Islander or Native Hawaiian	8 (6.1)	2 (5.4)	6 (6.4)
	White	49 (37.4)	12 (32.4)	37 (39.5)
	Other	2 (1.5)	1 (2.7)	1 (1.1)
	Declined to answer	3 (2.3)	1 (2.7)	2 (2.1)
**Marital status (total sample: n=121; Facebook: n=33; Reddit: n=88), n (%)**				
	Single	94 (77.7)	25 (75.8)	69 (78.4)
	Married	6 (5)	1 (3)	5 (5.7)
	Separated or divorced	7 (5.8)	2 (6.1)	5 (5.7)
	Living with partner	13 (10.7)	5 (15.2)	8 (9.1)
	Widow or widower	1 (0.8)	0 (0)	1 (1.1)

^a^Percentages do not add to 100 because participants were allowed to select more than one option.

**Table 2:** Justice system involvement, substance use, and treatment history.

			Total sample, n (%)	Facebook, n (%)		Reddit, n (%)
**Justice system involvement (past year^a^; total sample: N=131; Facebook: n=37; Reddit: n=94)**						
	Arrested	126 (96.9)		36 (97.3)	90 (96.8)	
	≥1 day in jail	84 (64.1)		33 (89.2)	51 (54.3)	
	≥1 day in prison	26 (20)		12 (33.3)	14 (14.9)	
	Probation	53 (40.5)		17 (45.9)	36 (38.3)	
	Electronic monitoring	37 (28.5)		5 (13.5)	32 (34.4)	
	Court-mandated living arrangement	17 (13.2)		3 (8.1)	14 (15.2)	
	Parole	22 (16.9)		8 (21.6)	14 (15.1)	
	Awaiting court proceeding	17 (13)		7 (18.9)	10 (10.6)	
	Recently convicted and waiting to serve sentence	6 (4.6)		2 (5.4)	4 (4.3)	
	Involved in collaborative court	15 (11.5)		5 (13.9)	10 (10.6)	
Hazardous alcohol use (total sample: n=104; Facebook: n=27; Reddit: n=77)			54 (51.9)	14 (51.9)		40 (51.9)
Hazardous drug use (total sample: n=115; Facebook: n=29; Reddit: n=86)			66 (57.4)	16 (55.2)		50 (58.1)
Current substance use treatment (total sample: n=125; Facebook: n=35; Reddit: n=90)			28 (22.4)	3 (8.6)		25 (27.8)
Current substance use treatment, mandated by court (total sample: n=28; Facebook: n=3; Reddit: n=25)			26 (96.3)	3 (100)		23 (95.8)
**Treatment type (total sample: N=131; Facebook: n=37; Reddit: n=94)**						
	Inpatient or residential	3 (2.3)		0 (0)	3 (3.2)	
	Partial hospitalization	5 (3.8)		0 (0)	5 (5.3)	
	Opioid replacement	9 (6.9)		1 (2.7)	8 (8.5)	
	Narcan or naloxone prescription	1 (0.8)		0 (0)	1 (1.1)	
	Narcan or naloxone use	0 (0)		0 (0)	0 (0)	
	Other medications	1 (0.8)		1 (2.7)	0 (0)	
	Individual counseling	14 (10.7)		3 (8.1)	11 (11.7)	
	Group counseling	12 (9.2)		2 (5.4)	10 (10.6)	
	Peer support	16 (12.2)		3 (8.1)	13 (13.8)	
	Other treatment	0 (0)		0 (0)	0 (0)	

^a^Percentages do not add to 100 because participants were allowed to select more than one option.

### Interest in Digital Health Interventions

Nearly all participants reported owning a smartphone (115/122, 94.3%) or having regular access to a computer (113/122, 92.6%). In total, 62.6% (82/131) of the participants expressed interest in participating in at least one digital health substance use intervention listed (asked as dichotomous yes or no). Each option presented to participants had relatively similar levels of interest (between 28/131, 24%, and 35/131, 27%, of the participants reported that they would consider participation). When asked to rank digital health substance use interventions, the three most popular were (1) receiving appointment reminders via SMS text messages, (2) web-based peer support community, and (3) private groups on Facebook focusing on reducing substance use. Those who reported hazardous substance use (Multimedia Appendix 1) reported more openness to motivational posts on social media (t_101_=−1.96; *P*=.05) and coaching by providers via SMS text messaging or secure messaging (t_101_=−2.55; *P*=.01) than their peers without hazardous substance use.

## Discussion

### Principal Findings

This study demonstrated the feasibility of recruiting a national sample of justice-involved young adults via social media. With some minor adjustments to initial advertisements and incentives, recruitment was efficient and cost-effective. Over the course of approximately 2 weeks, we surpassed our recruitment goal of 100 participants (the final sample included 131 participants) and spent US $1356 on advertisements. Reddit was somewhat more cost-effective than Facebook in recruiting participants (US $8.17 per participant vs US $15.88 per participant). Despite this cost difference, placing advertisements on multiple social media platforms was helpful to recruit a diverse sample of justice-involved young adults.

Although our sample was fairly heterogenous in terms of race and ethnicity, there were key racial and ethnic differences between the sample included in this study and the overall population of justice-involved adults in the United States. When compared with the overall population of adults who have been arrested in the past year, this study included a higher proportion of Asian, Pacific Islander or Native Hawaiian, and American Indian participants than expected. For example, although approximately 1.3% of the population of adults arrested in the past year are Asian [42], 20.6% (27/131) of the participants of this study identified themselves as Asian. Similarly, Native American or American Indian people comprise approximately 2.4% of the total population of arrestees [42] but represented 6.1% (8/131) of our sample. These data are consistent with prior research demonstrating that using social media to recruit participants may be an effective way to recruit specific populations of minoritized groups that are underrepresented in clinical research [28,29].

Notably, Black-identified people were underrepresented in our sample. Although nationally, 26.1% of adults arrested in the past year identify as Black or African American [42], less than 18% (23/131) of our sample did. Just under 20% (26/131) of our sample identified themselves as Latinx or Hispanic, which roughly corresponds to national estimates: 18.8% of adults arrested in the past year are reported to be Hispanic or Latino [42]. Only 37.4% (49/131) of our sample identified as White, which is substantially lower than expected. Across the United States, 69.9% of adults arrested in the past year are White. This finding may be a result of our recruitment strategy, which was focused on advertising to participants from more diverse, urban locations and did not include rural communities.

We found that an image of a White-appearing person in handcuffs received the most engagement and was the most cost-effective for recruitment (US $0.57 per click). Despite the fact that most people arrested in any given year are White [42], stereotypes in the United States associating people of color with the criminal justice system remain pervasive. A White-appearing person in handcuffs may have been unexpected and the most eye-catching of the images we trialed. However, our sample included fewer than expected Black participants, thus raising the possibility that this image may have been less engaging to Black young adults. Although academic research regarding structural racism in advertising remains somewhat limited, this finding is consistent with prior research from the field of marketing showing that advertisements featuring images of Black people are significantly more likely to engage Black young adults than images of people from other racial and ethnic groups [43].

Digital health SUD interventions were broadly acceptable to survey respondents, with nearly two-thirds (82/131, 62.6%) of the sample expressing interest in one or more potential programs. The most broadly acceptable intervention was SMS text reminders for appointments. Interventions focused on fostering supportive communities on the web, either with peer support or via private Facebook groups, were also popular.

Notably, this study was conducted before the COVID-19 pandemic. When social distancing protocols were widely implemented across the United States in March 2020, access to in-person SUD treatment and peer support communities (eg, Alcoholics Anonymous) drastically reduced [44,45]. Providing most behavioral health services via telehealth is feasible, particularly with regulatory changes made during the pandemic [46,47]; however, many SUD treatment models rely on groups and thus are more challenging to adapt to web-based formats and often have less group cohesion and treatment alliance [48]. Surveillance data suggest substance use has increased during the COVID-19 pandemic [49], particularly among young adults, thus widening the already-considerable gap between the number of people who would benefit from treatment and the number of people who receive it. Digital health interventions for SUDs may therefore be even more acceptable than reported in this study, and swift adoption by providers and systems is more important than ever before.

Consistent with prior studies [9,10,50], the results of this survey demonstrated a need for SUD treatment among justice-involved young adults. More than half of the sample reported alcohol use (54/104, 51.9%) or drug use (66/115, 57.4%) in the hazardous range. Less than one-fourth (28/125, 22.4%) of the sample were currently receiving treatment, the vast majority of which was court ordered. Notably, young adults who reported hazardous substance use expressed preferences for active engagement with providers in digital spaces, such as receiving motivational posts and coaching by providers via SMS text messaging or secure messaging. More justice-involved young adults may, therefore, benefit from substance treatment. Reaching justice-involved young adults through social media, with the ultimate goal of expanding available digital health interventions, may reduce barriers to entry for care, especially for young adults who have difficulty attending in-person sessions or have ambivalent engagement in treatment.

### Strengths and Limitations

This study has several notable strengths and limitations that can inform future research. The strengths include nationwide sampling and the heterogeneity of young adults recruited in terms of gender, race and ethnicity, and substance use. Obtaining perspectives from a variety of young adults allowed a more comprehensive (ie, nationwide survey) investigation of young adults’ perspectives regarding the potential feasibility and acceptability of substance use digital health interventions. The study supports the use of social media to recruit justice-involved young adults for research studies and provides insight into several potentially acceptable avenues for digital health interventions. The use of these approaches has the potential to expand access to treatment and promote health equity among justice-involved young adults.

A key limitation of this study, similar to most internet-based data collection, involves verifying respondent identity. We relied on self-report of justice involvement, so it is possible that some participants mischaracterized their experiences to gain study entry. Given the minimal incentive and that only participants who completed the full survey received the incentive, false identification of oneself as a justice-involved person seems less likely. To protect our respondents’ privacy, we did not track IP addresses, so it is possible that some respondents attempted to take the survey more than once; in any cases where the email was identical (this occurred in 2 cases), the duplicate (second response) was removed. When possible, tracking IP addresses in future studies could provide an additional method of preventing multiple entries from a single individual.

Our study relied on recruitment from only 2 social media platforms, so we did not reach justice-involved young adults who use other forms of social media. Facebook and Reddit are used by many young adults: approximately 23% of Facebook users are aged between 18 and 24 years [51], and 58% of Reddit users are aged between 18 and 29 years [52]. Furthermore, Facebook and Reddit both offer easy-to-use advertisement platforms for conducting research. Future studies should consider expanding recruitment to other social media platforms (eg, YouTube and Twitter, which are used by 95% and 42% of young adults, respectively [23]) to reach a wider range of justice-involved youth adults. Justice-involved young adults who do not have internet access were unable to participate in this study. However, as 96% of young adults in the United States own smartphones [53], the vast majority of young adults have some form of internet access.

Although our sample was mixed in terms of gender, race, ethnicity, and substance use history, we only enrolled English-speaking young adults. This excludes a portion of the justice-involved population in the United States and decreases generalizability. The inclusion of individuals who speak languages other than English may provide insight into different preferences for digital health interventions, which should be considered in future studies and in design of interventions to promote health equity.

Finally, we did not collect data on concerns that young adults may have with engaging in digital health interventions or in internet-based SUD treatment or whether young adults would prefer traditional services over digital health. Such concerns will be critical to understand and address in future work developing digital health interventions.

### Future Directions

Despite limitations, this pilot study suggests several promising directions for future research. First, future studies should continue to hone recruitment methods using social media to reach larger samples of justice-involved young adults. Expanding this research to new platforms such as YouTube and Twitter, in addition to refining advertisements, will be critical for reaching a representative sample of justice-involved young adults. Future work should also test the efficacy of advertisements using a wider variety of text options and images featuring young adults from other racial and ethnic backgrounds, particularly Black young adults.

This study demonstrates that social media is an effective tool to recruit justice-involved young adults into clinical research; future work can build on this finding to develop robust empirical support for substance use treatment interventions for this population for whom a dearth of interventions are currently available and used [6,10]. Young adults indicated that digital health interventions were broadly acceptable. Adjuncts to existing treatment, such as receiving appointment reminders via SMS text messages, are already broadly implemented in many medical systems and have been effective in enhancing connection with care [54]. Systems serving young adults in the justice system, such as community mental health centers and courts, should consider adopting this technology as well.

Young adults who reported hazardous substance use were also open to coaching via secure messages or texting treatment providers may wish to implement this into existing practices to increase treatment engagement and improve outcomes. Although there are barriers to widely implementing this mode of communication, particularly with regard to suicide risk management and difficulty receiving reimbursement [49,55], existing structures of treatment are not effective for many justice-involved young adults [4]. Adapting treatment to the modalities by which young adults typically communicate may enhance treatment engagement and outcomes.

Private Facebook groups, with daily posts and discussion moderated by trained clinical staff, have been shown to reduce cigarette smoking and alcohol use among diverse populations of young adults [26,33,56]. Young adults found such groups to be convenient and expressed that social support is particularly beneficial [57]. Aspects of this model may be adapted for young adults involved in the justice system who seek to reduce their own substance use.

### Conclusions

This pilot feasibility study established the utility of social media in recruiting a broad sample of young adults involved in the criminal justice system. These young adults reported high rates of hazardous substance use; however, few were in treatment voluntarily. As this high-need, underserved population reported strong interest in digital health interventions, future work will leverage social media to create programs to engage justice-involved young adults in substance use treatment.

## Appendix

Multimedia Appendix 1 Acceptable digital mental health interventions for participants with and without hazardous drug and alcohol use. **P*=.05. ***P*=.01.

## Abbreviations

AUDIT: Alcohol Use Disorders Identification Test
DUDIT: Drug Use Disorders Identification Test
REDCap: Research Electronic Data Capture
SUD: substance use disorder
