# Supplementary figures and images for: Using Social Media to Engage Justice-Involved Young Adults in Digital Health Interventions for Substance Use: Pilot Feasibility Survey Study
Source: JMIR Form Res. 2022 Dec 2;6(12):e37609. doi: 10.2196/37609 (PMC9758636; doi:10.2196/37609)

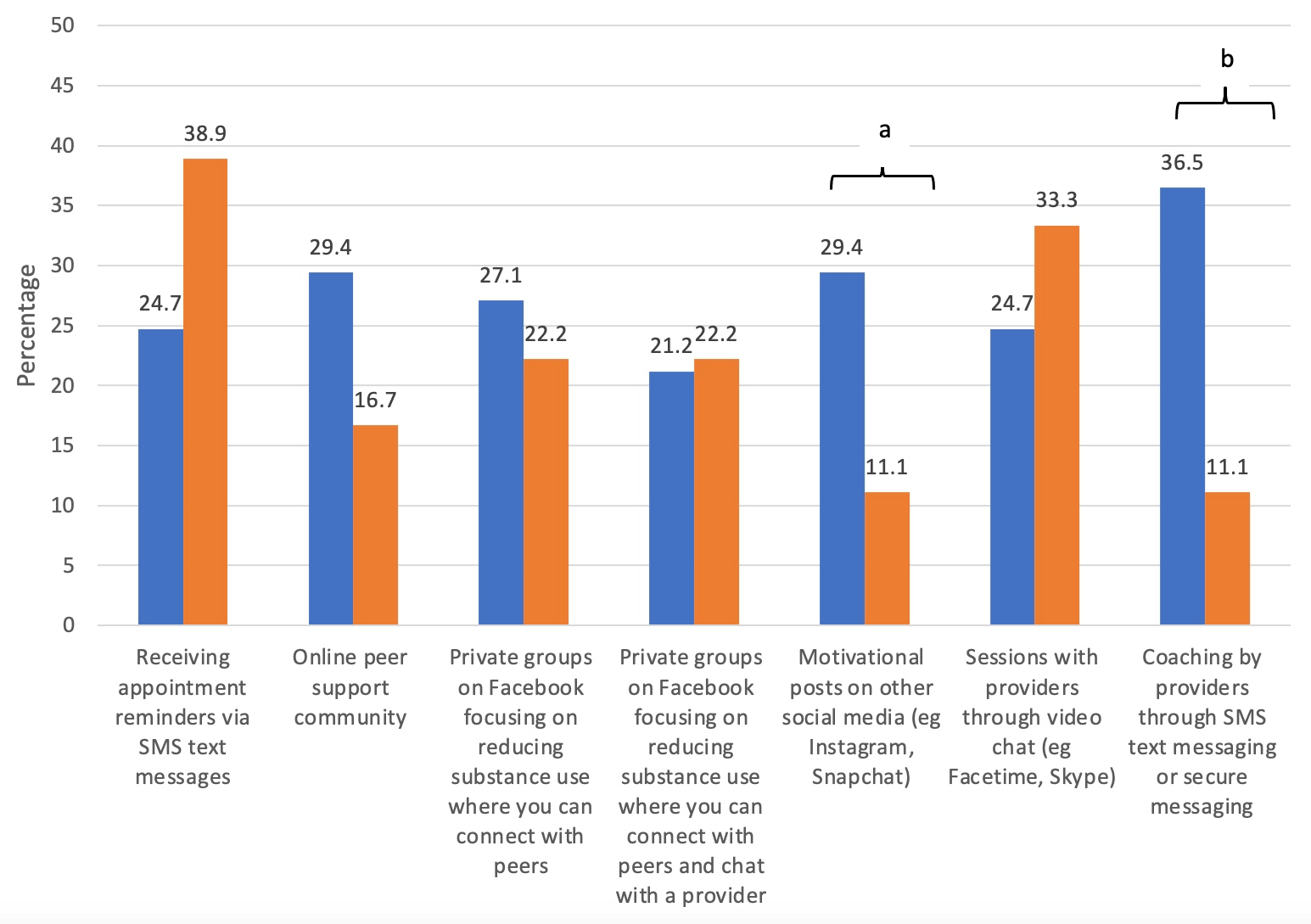

Supplement: Multimedia Appendix 1 [file formative_v6i12e37609_app1.png]
